# Smoked-Derived Volatile Phenol Analysis in Wine by Stir Bar Sorptive Extraction-Gas Chromatography-Mass Spectrometry

**DOI:** 10.3390/molecules26185613

**Published:** 2021-09-16

**Authors:** Ruiwen Yang, Armando Alcazar-Magana, Yanping L. Qian, Michael C. Qian

**Affiliations:** 1 Department of Food Science and Technology, Oregon State University, Corvallis, OR 97331, USA; maomaoyrw@163.com (R.Y.); alcazara@oregonstate.edu (A.A.-M.); yan.ping.qian@oregonstate.edu (Y.L.Q.); 2 College of Food Science and Engineering, Jilin University, Changchun 130062, China

**Keywords:** smoke-exposed wine, volatile phenols, SBSE-GC-MS, EG-PDMS

## Abstract

Smoke-derived taint has become a significant concern for the U.S. wine industry, particularly on the west coast, and climate change is anticipated to aggravate it. High volatile phenols such as guaiacol, 4-methylguaiacol, 4-ethylguaiacol, 4-ethylphenol, and *o*-, *p*-, *m*-cresols have been suggested to be related to smoke-exposed grape and wine. This paper describes an analytical approach based on ethylene glycol/polydimethylsiloxane (EG/PDMS)-stir bar sorptive extraction-gas chromatography-mass spectrometry (SBSE-GC-MS) to quantify or estimate the concentrations of some smoke-related volatile phenols in wines. Correlation coefficients with R^2^ ≥ 0.990 were obtained. This method can quantify most smoked-related volatile phenols down to 0.5 μg/L in wine in selective ion monitoring mode. Recovery for the targeted volatile phenols ranged from 72.2% to 142.4% in the smoke-tainted wine matrix, except for 4-vinylguaiacol. The standard deviations of the volatile phenols were from 0 to 23% in smoke-tainted wine. The approach provides another tool to evaluate wine smoke exposure and potential smoke taint.

## 1. Introduction

The west coast of the United States is an important wine-producing region for premium wines. However, the industry is vulnerable to wildfires that have elevated during the past decade. When grapes and grapevines are exposed to wildfire smoke, smoke compounds can be absorbed and stored in grapes as free aglycon or in glycosidic bound forms. Depending on the extent of smoke exposure, smoke-exposed grapes may produce smoke-tainted wines described as smoky, burnt, ash, smoky bacon, medicinal, and ashtray undesirable sensory characters [1,2], resulting in considerable economic losses. Numerous studies have been conducted to address smoke taint in wine, including vineyard and grape treatments to minimize the taint [3,4]. Still, the smoke taint issue has not been solved. Many volatile compounds are present in smoke. Guaiacol has been considered one of the essential compounds in smoke-tainted wines because of its low aroma threshold and high content after smoke exposure [5]. In addition, 4-methylguaiacol, syringol, 4-methylsyringol, *o*-, *p*-, and *m*-cresols have been suggested as indicators for smoke-tainted wines [6]. However, it is not exactly clear which compounds contribute to this off-aroma.

Various analytical methods have been developed to determine smoke taint volatiles, and each has its advantages and limitations. The gas chromatography-mass spectrometry (GC-MS) method with stable isotope dilution assay (SIDA) is the most widely used technique for the analysis. However, due to the low concentration of smoke-related compounds in wine and complex wine matrices, the analytes need to be extracted and enriched before GC-MS analysis. Kennison et al. [1] used liquid-liquid extraction-based GC-MS with SIDA methods to identify guaiacol, 4-methylguaiacol, 4-ethylguaiacol, 4-ethylphenol, eugenol, and furfural in wines made from smoked grapes. Fudges and coworkers [3,4] used a similar approach to quantify concentrations of smoke-related compounds before and after treatments on smoke-tainted wines. They found that reverse osmosis and solid-phase adsorption reduced the concentrations of volatile phenolic compounds, and some refining agents could reduce smoke taint in wines, particularly activated carbon. Pollnitz et al. [7] compared liquid-liquid extraction and solid-phase microextraction (SPME), combined with GC-MS to analyze guaiacol, 4-methylguaiacol, 4-ethylphenol, 4-ethylguaiacol, *trans*, *cis*-oak lactone, and vanillin in oak extracts. The results indicated that both methods were rapid and robust, but SPME is more convenient and straightforward. Although the SPME technique is quick and sensitive, other volatile compounds in the wine matrix could compete for active sites on the fibers affecting the quantification [8]. Thus, stable isotope-labeled chemicals must be used for all compounds for reliable quantification. In addition, the SPME fiber has a limited capacity for volatile extraction. Stir bar sorptive extraction is another technique to extract volatiles from aqueous solutions, it relies on a polymer (typically polydimethylsiloxane (PDMS)) coated on a stir bar to extract the volatiles from samples and then desorb the volatiles onto GC-MS for analysis. The stir bar has much more polymer loading than the SPME fiber, thus can absorb more compounds for improved sensitivity. Berrou et al. [9] compared the SPME with the SBSE/headspace sorptive extraction (HSSE) method combined with GC-MS to analyze volatile compounds produced by *Staphylococcus aureus*. The results showed that SBSE/HSSE possesses higher concentration capacities and greater sensitivity than SPME. The SBSE-GC/MS method has been widely used for volatile compounds analysis in food and beverages. Du et al. [10] studied volatile compounds from Marion blackberry using PDMS-based SBSE-GC/MS. Song et al. [11] investigated free and bound volatile compounds in Merlot grape using PDMS-based SBSE-GC/MS. PDMS-based SBSE has been applied in many other fields. The PDMS-stir bar is robust and has a low affinity to ethanol. It is particularly suitable for volatile analysis in alcoholic beverages, such as wine.

However, the PDMS stir bar lacks sensitivity for polar compounds. Ethylene glycol/silicone (EG) stir bar compensates the PDMS stir bar thanks to the polar nature of the phase. The EG-based stir bar can be used alone for polar compounds analysis or combined with the PDMS-based stir bar to analyze both polar and non-polar compounds. Ochiai et al. [12] investigated the odor compounds in roasted green tea using combined PDMS and EG-silicone-based SBSE-GC/MS. The results showed that compared with conventional SBSE (using PDMS or EG alone), the combined SBSE method with both EG and PDMS stir bars was more effective in enriching most compounds. For targeted polar compound analysis, the EG-based SBSE is an ideal choice. However, the application of EG-based SBSE is more challenging for alcoholic samples because the extraction principle of analytes on EG-stir bar involves H-bonding; thus, alcohol, carboxy acids, and the pH will affect the extraction of the analytes. Zhou et al. [8] developed an EG/PDMS copolymer-based SBSE-GC-MS method to analyze 4-ethylphenol, 4-vinylphenol, 4-ethylguaiacol, and 4-vinylguaiacol in alcoholic beverages, and good sensitivity and reproducibility were achieved for these compounds. However, as far as we know, there is no EG/PDMS copolymer-based SBSE-GC-MS method for the targeted analysis of other smoke-tainted volatile biomarker compounds in wine. This study focuses on the smoke-related volatile phenol analysis in wine using EG/PDMS SBSE to enhance quantification limits.

## 2. Results

### 2.1. Establishment of the Standard Calibration Curve

**Figure 1:**
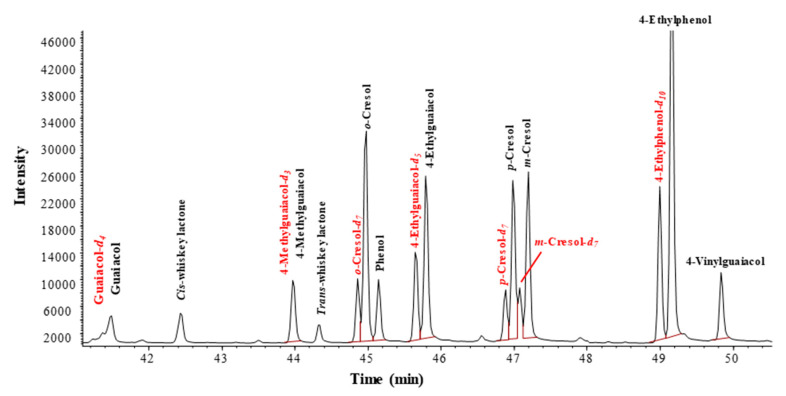
Chromatogram of eleven targeted compounds and internal standards under the chromatographic condition.

Standard calibration curves showed a good linear relationship (R^2^ > 0.99, Table 1). The injection range of 4-methylguaiacol and phenol was from 0.5 μg/L to 50 μg/L; the injection range of the remaining volatile compounds was from 0.5 μg/L to 37.5 μg/L. Eleven targeted compounds and internal standards were well-separated under this chromatographic condition (Figure 1). Final concentrations in the chromatogram for labeled and non-labeled standards were 5 μg/L and 12.5 μg/L, respectively.

### 2.2. Estimation of Volatile Phenols in Wines by EG/PDMS Based SBSE-GC-MS

The analytical results of SBSE-GC-MS (Table 2) showed the concentrations of guaiacol, *cis*-whiskey lactone, cresols, phenol, 4-ethylguaiacol, 4-ethylphenol, and 4-vinylguaiacol were at higher levels in the smoke-tainted wine, especially guaiacol, cresols, and phenol. The concentration of guaiacol was 33.6 ± 6.2 μg/L in the smoke-tainted wine versus 15.9 ± 3.5 μg/L in the control wine. Similarly, the concentration of *o*-cresol was 5.85 ± 0.4 μg/L and 1.92 ± 0.52 μg/L in the smoke-tainted and the control wine respectively; *p*-cresol was 4.07 ± 0.38 μg/L and 2.78 ± 0.63 μg/L, respectively; *m*-cresol 5.08 ± 0.49 μg/L and 1.75 ± 0 μg/L, respectively; phenol was 26.5 ± 6.19 μg/L and 0.65 ± 0 μg/L, respectively. The concentrations of the other volatile compounds were similar for both wines.

### 2.3. Recovery and Precision of the Analytical Method

We studied the recovery of the volatile phenols in the smoke-tainted wine by spiking the wine sample with the 11 phenolic compounds at a concentration of 5 μg/L (Table 2). The results showed that guaiacol had a good recovery (99%). The recoveries of *cis*-whiskey lactone (142%), 4-methylguaiacol (131%), *trans*-whiskey lactone (120%), cresols (132%, 134% and 121%), 4-ethylguaiacol (114%), and 4-ethylphenol (134%) were over 100%. The phenol recovery was only 72%, and 4-vinylguaiacol was below 50%. This method’s precision was studied by measuring the same sample three times for the control and smoke-tainted wines (Table 2). The coefficient of variation (CV) value of volatile compounds in control wine was below 10% for most compounds, except guaiacol (22%), *o*-cresol (27%), *p*-cresol (23%), and 4-ethylphenol (38%). In the case of the smoke-tainted wine, the CV value of volatile compounds was also below 10% for most compounds, except guaiacol (19%), phenol (23%), and 4-ethylphenol (16%). These variations are probably due to the variation of the stir bar. These results suggested that the EG-PDMS stir bar technique can be used to estimate the concentration of many smoke-related volatile phenol compounds in wine, except for 4-vinylguaiacol. More precise control in sample preparation and stir bar usage life needs to be evaluated.

## 3. Discussion

The results showed that the concentrations of guaiacol, cresols, and phenol in the smoke-tainted wine sample were higher than those in the control wine, suggesting they may be related to smoke taint in wine. However, more smoke-exposed wines need to be analyzed to verify if these compounds can be used as indicators for smoke exposure. In addition, the actual smoke-tainted compounds have not been entirely identified. Hence, more smoke-related compounds should be thoroughly investigated.

Notably, volatile phenolic compounds’ concentrations in the smoke-tainted wine sample were lower than their sensory detection thresholds in wine. The detection threshold of guaiacol, 4-methylguaiacol, phenol, and 4-vinylguaiacolin in red wine is 75 μg/L, 65 μg/L, 25,000 μg/L, and 380 μg/L, respectively [13]. Although the concentration of phenol in smoke-tainted wine was 40 times higher than the control wine, the sensory threshold of this compound in wine is very high. The ratio of concentration to the sensory threshold (odor activity value) is only 0.01, suggesting phenol is unlikely a smoke contributor. Similarly, 4-vinylguaiacol is unlikely a smoke contributor in this wine. The detection threshold of *cis*-whiskey lactone, 4-ethylguaiacol, and 4-ethylphenol in red wine of cis-whiskey lactone, 4-ethylguaiacol, and 4-ethylphenol is 74 μg/L, 110 μg/L, and 605 μg/L, respectively [14]. The concentrations of *cis/trans*-whiskey lactones were similar in the smoke taint wine sample and the control wine sample, suggesting they are unrelated to smoke exposure. The detection threshold of *m*-cresol, *p*-cresol, and *o*-cresol in red wine is 20 μg/L, 64 μg/L, and 62 μg/L, respectively [15]. Although the compounds analyzed were much lower than their sensory thresholds, the smoke-tainted wine sample had a distinct smoky aroma. Likely, other smoke compounds may also contribute to the smoky aroma of the wine. In addition, the flavor additive effect may also contribute to the smoke-tainted wine sample. A similar situation was observed by Wilkinson et al. [1] and Fudge et al. [1,4]. Wilkinson et al. [1] reported that guaiacol and 4-methylguaiacol concentration was 28 μg/L and 7 μg/L, respectively. They also speculated that neither guaiacol nor 4-methyl guaiacol was the only cause of the smoky odor. Mayr et al. [16] suggested that a variety of volatile phenols together contributed to the smoky odor. Volatile phenol compounds with concentrations below their threshold may be contributing synergistically to the smoky aroma providing a new challenge to unravel the analysis of smoky compounds. Nevertheless, identifying these additional smoke compounds and understanding the synergistic effect of the volatile phenols are of active ongoing research, which is beyond the scope of this study.

The recovery was studied with the volatile phenols spiked at 5 μg/L levels in the wine. This concentration level was low but comparable with the concentrations in the unsmoked wines. Liu et al. [17] studied the recovery of a liquid-liquid extraction-based GC-MS/MS method for volatile phenol analysis in smoke-tainted grapes. They spiked a mixture of standards at a concentration of 100 ng/g, which is 20 times what we added. Even at that high level, a relatively low recovery (<80%) was observed for most volatile phenol compounds in their research, and the recovery for 4-ethylphenol in different wines ranged from 19.5 ± 0.5% to 51.4 ± 2%. Similar results were also found by Noestheden et al. [18] when the researchers spiked the samples at 100 ng/g level, and the accuracy and CV ranged from 67 to 124% and from 2 to 14%, respectively. Noestheden et al. [18] attributed the low recoveries of some compounds to the co-eluting matrix interference and inappropriate internal standard. For example, Noestheden et al. used d_4_-4-ethylphenol as the internal standard for the quantitative analysis of *o*-cresol. All of these researches have demonstrated the challenges with volatile phenol analysis. The proposed EG-PDMS-SBSE method had relatively good recovery at lower-spiked concentrations for most volatile phenols, with reasonable accuracy, except 4-vinylguaiacol, with only 32% recovery.

This EG/PDMS-SBSE-GC-MS approach was developed for smoke-related volatile phenol analysis based on the previous method, with many improvements and modifications [8]. First, the research focused on 11 volatile phenol compounds relative to smoke-tainted compounds. Therefore, the corresponding separation parameters were optimized, including the temperature program, column type, and column flow rate. Then, this method improved the linear range of the analytes. The lower end of the linear range in this method was 0.5 μg/L, which provides relatively more accurate quantification at low concentrations for most compounds, which is essential for smoke analysis when the wine samples need to be diluted to eliminate the matrix interference. Besides, 12% ethanol was adopted to establish the calibration curve because most wines have an alcohol range from 8% to 15%. Finally, to avoid the co-eluting matrix interference, the stable isotope compounds were used as internal standards in this method. This method provided another approach for studying volatile phenol compounds in smoke-exposed wine. Further research will apply this method to assess the smoke exposure on wine quality, including total volatile phenol analysis after acid hydrolysis, to study the release of volatile phenolic compounds from glycosides.

## 4. Materials and Methods

### 4.1. Chemicals and Reagents

Standards of guaiacol (≥98%), 4-methylguaiacol (99%), whiskey lactone (≥98%), *o*-cresol (≥99.5%), phenol (≥99.5%), *p*-cresol (≥99%), *m*-cresol (99%), 4-ethylphenol (≥98%) and 4-vinylguaiacol (≥98%) were obtained from Sigma-Aldrich (St. Louis, MO, USA). 4-Ethylguaiacol (≥98%) was obtained from Alfa Aesar (Heysham, Lancashire, UK). Isotope internal standards, 2-methoxyphenol-3, 4, 5, 6-d_4_ (guaiacol-d_4_; 98.5%-d_4_), 2-methoxy-4-methylphenol-3, 5, 6-d_3_ (4-methylguaiacol-d_3_; 99.3%-d_3_), 4-ethyl-d_5_-2-methoxyphenol (4-ethylguaiacol-d_5_; 99.7%-d_5_), 4-ethylphenol-d_10_ (99.2%-d_10_), *m*-cresol-d_7_ (98.7%-d_7_), *p*-cresol-d_7_ (99.2%-d_7_), and *o*-cresol-d_7_ (99%-d_7_) were purchased from CDN Isotopes (Pointe-Claire, QC, Canada).

Tartaric acid (A.R. grade) was bought from A Johnson Matthey Company (Heysham, Lancs, UK). Ethanol (HPLC grade) was obtained from Greenfield Global USA Inc. (Brookfield, CT, USA). Methanol (HPLC grade) and sodium hydroxide 10 N solution (30% *w*/*w*) were purchased from Fisher Scientific (Fair Lawn, NJ, USA). Potassium phosphate dibasic trihydrate (K_2_HPO_4_·3H_2_O, ≥99%) and potassium phosphate (KH_2_PO_4_, 99.0%) were obtained from Acros organics (Carlsbad, CA, USA). Milli-Q water was obtained from a Milli-Q purification system (Millipore, Boston, MA, USA).

An internal standard solution (IS) was made by dissolving guaiacol-d_4_, 4-methylguaiacol-d_3_, *o*-cresol-d_7_, 4-ethylguaiacol-d_5_, *p*-cresol-d_7_, *m*-cresol-d_7,_ and 4-ethylphenol-d_10_ in absolute methanol and diluted to a final concentration of 10 mg/L.

### 4.2. Sample Preparation

The smoke-tainted wine sample was obtained from a local winery that was affected by the 2015 fire. The control wine was not smoke-tainted from the region. Four milliliters of sample wine were diluted with 16 mL of phosphate buffer (1 M, pH 7) in a 20 mL glass vial. Ten microliters of IS were added to obtain a final concentration of 5 μg/L in solution. Phosphate buffer (1 M, pH 7) was made by mixing 1 M K_2_HPO_4_ solution and 1 M KH_2_PO_4_ solution to reach the required pH. An ethylene glycol (EG)/polydimethylsiloxane (PDMS) copolymer (1 cm length, 0.5 mm thickness, Gerstel, Inc., Linthicum, MD, USA.) was introduced into the vial for the extraction. The extraction was three hours at the stirring speed of 1000 rpm at room temperature [8]. The stir bar was rinsed with Milli-Q water, dried with Kimwipe tissue after extraction, and then placed in a sample holder for further GC-MS analysis.

### 4.3. SBSE-GC-MS Analysis

The SBSE-GC-MS analysis was performed on an Agilent 7890 GC-5975 MSD system (Agilent Technologies, Inc., Santa Clara, CA, USA) equipped with a multi-purpose sampler (MPS) (Gerstel, Inc.,). The sample holder was put into the thermal desorption unit (TDU) with a splitless mode. The initial temperature of TDU was 35 °C, then increased to 220 °C at a rate of 120 °C/min, and further maintained for 3 min. Then, the volatile compounds reached a programmed temperature vaporizing (PTV) injector. The pressure was 26 psi, and purge flow to split vent was 50 mL/min at 4 min. The compounds were trapped in the cooled injection system (CIS-4) at −80 °C with liquid nitrogen. Then, the CIS was heated at 10 °C/s to reach 220 °C and held for 3 min. Separation was achieved on a ZB-WAX column (60 m × 0.25 mm ID, 0.5 μm film thickness, Phenomenex, Torrance, CA, USA). The initial temperature was 40 °C, held for 4 min, then increased to 230 °C at a rate of 4 °C/min and maintained for 10 min. The total run time was 61.5 min, with the helium flow rate at 1.5 mL/min. Mass spectrometric detection was performed in selective ion monitoring (SIM) mode. The MS transfer line and ion source temperature was 280 °C and 230 °C, respectively. Selective mass ions were used to quantify the volatile phenols. Four groups were set. Selected ion of group one started from 0 min. Selected ions of group two started from 39 min (guaiacol, *cis*-whiskey lactone, 4-methylguaiacol, and *trans*-whiskey lactone). Selected ions of group three started from 44.5 min (*o*-cresol, phenol, 4-ethylguaiacol, *p*-cresol, and *m*-cresol). Selected ions of group four started from 48 min (4-ethylphenol and 4-vinylguaiacol).

### 4.4. Method Development

#### 4.4.1. Standard Calibration Curve

Appropriate amounts of guaiacol, 4-methylguaiacol, whiskey lactone, *o*-cresol, phenol, *p*-cresol, m-cresol, 4-ethylphenol, 4-ethylguaiacol, and 4-vinylguaiacol were mixed into 10 mL absolute methanol and reached a final concentration of 100 mg/L. Then, 1 mL 100 mg/L solution was diluted by 9 mL methanol to obtain a 10 mg/L concentration stock solution and was stored at −4 °C. The synthetic wine was made by dissolving 3.5 g tartaric acid in 1 L 12% ethanol solution. The pH was adjusted to 3.5 using sodium hydroxide 10 N solution. Four milliliters of synthetic wine were diluted with 16 mL of phosphate buffer (1 M, pH 7) in a 20 mL glass vial. An aliquot of 10 μL of IS was then added. The standard calibration curve was prepared using the stock solution, ranging from 1 mg/L to 100 mg/L. Fifty μL of the standard calibration solutions were also added to the vial, respectively, to obtain a final concentration range from 0.5 μg/L to 50 μg/L in 20 mL solution (0.5 μg/L, 5 μg/L, 12.5 μg/L, 25 μg/L, 37.5 μg/L, and 50 μg/L). The standard solution was analyzed using SBSE-GC-MS as previously described. Selected ions were used to build the calibration curve by Chemstation software (Agilent Technologies, Inc.).

#### 4.4.2. Method Reproducibility

The GC-MS results were calculated using Chemstation software (version E.02.01, Agilent Technologies, Inc.). Reproducibility was achieved by analyzing the samples in triplicates. The results were expressed as the mean ± standard deviation (SD), and the CV was also calculated.

#### 4.4.3. Recovery Calculation

The smoke-tainted wine sample was used to measure the recovery of all targeted compounds. The concentration of 11 compounds (C_1_) in the sample was analyzed, then the wine sample was spiked with 5 μg/L of 11 compounds (C_2_). The spiked sample was then analyzed using the SBSE-GC-MS method. The recovery was calculated from the following equation:

Recovery(%) = ((C_2_ − C_1_)/5 μg/L) × 100%. (1)

## 5. Conclusions

In this study, the ethylene glycol-polydimethylsiloxane-based stir bar sorptive extraction-gas chromatography-mass spectrometry (EG/PDMS-SBSE-GC-MS) technique was assessed for analyzing smoke-related volatile phenol compounds in wines with stable isotope-labeled compounds as internal standards. Good linearities were obtained for all compounds from 0.5 to 37.5 ug/L, covering the normal concentration ranges of the compounds present in wine. This method was used to quantify or estimate the concentrations of some volatile phenol compounds in a smoke-tainted and a non-smoked wine. It was found that the smoke-tainted wine contained higher concentrations of phenol, *o*-cresol, and guaiacol than the control wine. However, all the smoke-associated compounds analyzed were below the sensory detection thresholds, suggesting the possibility of synergistic effects among these compounds or other unidentified smoke-contributing compounds in the smoke-tainted wine sample. This manuscript is the first attempt to use the EG/PDMS-GC-MS approach to analyze smoke-related volatile phenols in wine, and it provides an alternative tool for smoke exposure research.

## Figures and Tables

**Table 1:** Internal standard (IS), retention time (RT), target ions, calibration equations, standard range, linearly dependent coefficient (R^2^) of volatile compounds in smoke-tainted wines using SBSE-GC-MS.

Compound	Quantify Ion	Qualify Ions	Equation	Linear Range (μg/L)	R^2^
Guaiacol-d_4_	128	113			
Guaiacol	124	109	y = 0.02675x^2^ + 1.033x + 0.7798	0.5–37.5	0.9901
*Cis*-whiskey lactone	99	71	y = 3.006x − 0.5691	0.5–37.5	0.9968
4-Methylguaiacol-d_3_	141	126			
4-Methylguaiacol	138	123	y = 0.757x + 0.005532	0.5–50	0.9995
*Trans*-whiskey lactone	99	69	y = 0.3896x − 0.008664	0.5–37.5	0.9995
*o*-Cresol-d_7_	115	113			
*o*-Cresol	108	107	y = 1.097x − 0.02329	0.5–37.5	0.9996
Phenol	94	66	y = 0.03047x^2^ + 0.3045x + 0.7194	0.5–50	0.9990
4-Ethylguaiacol-d_5_	157	139			
4-Ethylguaiacol	137	152	y = 0.661x − 0.08313	0.5–37.5	0.9990
*p*-Cresol-d_7_	115	113			
*p*-Cresol	108	107.77	y = 1.025x − 0.05198	0.5–37.5	0.9993
*m*-Cresol-d_7_	115	113			
*m*-Cresol	108	107.79	y = 1.12x + 0.1263	0.5–37.5	0.9991
4-Ethylphenol-d_10_	131	113			
4-Ethylphenol	107	122	y = 3.432x − 0.1754	0.5–37.5	0.9992
4-Vinylguaiacol	150	135	y = 0.3353x + 0.05101	0.5–37.5	0.9984

**Table 2:** Quantification results of smoke-tainted wine and control wine, CV and recovery.

Compounds	Control (μg/L)	CV (%)	Smoke-Tainted (μg/L)	CV (%)	Recovery
Guaiacol	15.9 ± 3.5	22	33.6 ± 6.2	19	99%
*Cis*-whiskey lactone	17.6 ± 1.7	10	19.1 ± 1.0	5	142%
4-Methylguaiacol	6.63 ± 0.21	3	6.37 ± 0.25	4	131%
*Trans*-whiskey lactone	53.1 ± 4.4	8	41.1 ± 3.6	9	120%
*o*-Cresol	1.92 ± 0.52	27	5.85 ± 0.40	7	132%
Phenol	0.65 ± 0.00	0	26.5 ± 6.2	23	72%
4-Ethylguaiacol	3.80 ± 0.25	7	3.82 ± 0.20	5	114%
*p*-Cresol	2.78 ± 0.63	23	4.07 ± 0.38	9	134%
*m*-Cresol	1.75 ± 0.00	0	5.08 ± 0.49	10	121%
4-Ethylphenol	2.38 ± 0.90	38	2.45 ± 0.39	16	134%
4-Vinylguaiacol	0.35 ± 0.00	0	2.10 ± 0.00	0	32%

The results were expressed as the mean ± standard deviation (SD). Besides, CV means coefficient of variation.

## Data Availability

The data presented in this study are available on request from the corresponding author.

## Sample Availability

Samples of the compounds are available on request from the corresponding authors.
